# Supplementary material for: Pesticide Use Practices in Root, Tuber, and Banana Crops by Smallholder Farmers in Rwanda and Burundi
Source: Int J Environ Res Public Health. 2019 Jan 31;16(3):400. doi: 10.3390/ijerph16030400 (PMC6388262; doi:10.3390/ijerph16030400)
Supplement: Supplementary file 1 [file ijerph-16-00400-s001.pdf]

Questionnaire: Farmer's perception of the impact of pests and diseases on potato, sweetpotato, banana and cassava

Part A: Interview background Questionnaire No……………………………………..

1. Which of these crops is this household producing during this 2nd cropping season 2014 B? (Yes/No)
   (i) potatoes ………………… (ii) sweetpotatoes…………….……… (iii) bananas/plantain……………………..….. (iv) cassava …………………………………….
2. Respondent's name:……………………………….......................Mobile No(s):……………………………………………………………….
3. Date of interview:……………………………….Interviewed by:……………………………………Checked by:………………………..
4. Country:…………………………Province:…………………….....District:……….……..………Sector:……………….……Cell:…………….………Village……………
5. Latitude:………………………………..Longitude:………………………………………Elevation (m):…………………………

Part B: Current household composition and Characteristics

How many people do live in this household during this cropping season?

Children (0-5yrs)…………Children (6-17)……….Men (18-65)...............Women (18-65).................Elderly men (≥ 65 yrs)…......... Elderly women (≥ 65 yrs)………….

Name (Please give the details of both the husband and the wife for each column in this table)	Sex	Relationship to HH head Codes A	Marital status Codes B	Age (years)	Formal Education		Occupation Codes D		Annual income (RWF/BIF)	
					Codes C	School Years	Main	Second	Farm	Off-farm
…………………………………………(HH head)										
……….…………………………………(Spouse)										

Codes A		Codes B	Codes C	Codes D	
1. HH head	8. Son in-law	1. Married living with spouse	1.None	0.None	7. Household chores
2. Spouse	9. Daughter in-law	2. Married but spouse away	2. Primary	1. Farming (Crop+Livestock)	8. Handcraft/weaving
3. Hired worker	10. Grand child	3. Divorced/Separated	3. Secondary Ordinary	2. Salaried employment	9. Other (Specify)…………………………………….
4. Son	11. Other relative	4. Widow/Widower	4. Secondary Advanced	3. Retail business	………………………………………………………………………
5. Daughter	12. Other (specify)	5. Never married	5. Diploma after O'level	4. Casual labourer on-farm	……………………………………………………………
6. Brother		6. Other (Specify)	6. Diploma after A level	5. Casual labourer off-farm	
7. Sister			7. University	6. School/college child	

Prepared by: Joshua OKONYA

Part C: Crop production constraints

What are your **main** problems in the production and marketing of the potato, sweetpotato, banana and cassava?

Give scores (3=most important, 2=moderate, 1=very little importance, 0=not a problem)

**Production, marketing and other factors**	**Score (potato)**	**Score (sweetpotato)**	**Score (banana)**	**Score (cassava)**
Insect pests				
Diseases				
Nematodes				
Weeds				
Low soil fertility				
Poor quality planting material				
Drought (unpredictable rainfall)				
High cost of planting material				
High cost of fungicides				
High cost of insecticides				
High cost of fertilizers				
Low market prices				
High cost of transport				
High cost of Nematicide				
High cost of herbicides (weed control)				
Exploitation by brokers				
Short shelf life (perishability)				
Floods/mud slides/ soil erosion				
Vertebrate pests (wild &domestic animals, rats)				
Invertebrate pests (millipedes, nematodes)				

Prepared by: Joshua OKONYA

Part D: Farmers knowledge of insect pests of potato, sweetpotato, banana and cassava

Do you know of any insect pest which attack **potato, sweetpotato, banana and cassava**? If yes, give pest name, appearance & type of damage

Crop	No	Insect pest (Local name)	Insect pest (English name)	Description of pest features	Type of damage caused
**Potato**	1				
	2				
	3				
	4				
	5				
**Sweetpotato**	1				
	2				
	3				
	4				
	5				
**Banana**	1				
	2				
	3				
	4				
	5				
**Cassava**	1				
	2				
	3				
	4				
	5				

Can you identify any of these insect pests? Show the farmer pictures or preserved samples of insects and related damage

Crop	Insect pest	Insect pest (local name)	Damage level (3.Very severe, 2.Moderate, 1.Very low)
**Potato**	Potato tuber moth/		
	Leaf miner fly		
	Aphids		
	Cutworm		
	Whiteflies		
	Ants (wire, fire, red)		
	Other (Specify)		
**Sweetpotato**	Sweetpotato weevils		
	Sweetpotato butterfly		
	Sweetpotato armyworm		
	Sweetpotato hornworm		
	Whiteflies		
	Clear wing moth		
	Other (Specify)		
**Banana**	Banana weevil		
	Banana nematodes		
	Other (Specify)		
**Cassava**	Whiteflies		
	Green mites		
	Aphids		
	Mealybugs		
	Other (Specify)		

Part E: Farmers knowledge of diseases of potato, sweetpotato, banana and cassava

Do you know of any disease which attack **potato, sweetpotato, banana and cassava**? If yes, give disease name and sign

Crop	No	Disease (English name)	Disease (Local name)	Type of damage caused to the plant (sign)
**Potato**	1			
	2			
	3			
	4			
	5			
**Sweetpotato**	1			
	2			
	3			
	4			
	5			
**Banana**	1			
	2			
	3			
	4			
	5			
**Cassava**	1			
	2			
	3			
	4			
	5			

Can you identify any of these diseases? Show the farmer pictures of disease signs

**Crop**	**Disease (English name)**	**Disease (local name)**	**Damage level** (3.Very severe, 2.Moderate, 1.Very low)
**Potato**	Bacterial wilt		
	Late blight		
	Early blight		
	Viral diseases		
	Fusarium wilt		
	Other (Specify)		
**sweetpotato**	Sweetpotato virus disease (SPVD)		
	Alternaria leaf blight		
	Root rots		
	Other (Specify)		
	Other (Specify)		
**Banana**	Banana bacterial wilt		
	Fusarium wilt		
	Bunchy top disease		
	Black sigatoka disease		
	Other (Specify)		
**Cassava**	Cassava mosaic disease (CMD)		
	Cassava brown streak virus disease (CBSD)		
	Cassava bacterial blight (CBB)		
	Other (Specify)		

Part F: Pest and disease management in potato

Which of the following control measures do you use in pest management?

Crop	Control method	Used (Yes/No)	Product name (Read it from the pesticide container)	Active Ingredient (Read it from the pesticide container)	Target pest or disease	Application freq. per season (Min & Max times, give range)	Quantity (mls/kg per Knapsack)	Knap-sack size (L)	Area sprayed (s)
**Potato**	Chemical insecticides		1.						
			2.						
	Chemical fungicides		1.						
			2.						
	Chemical herbicides		1.						
			2.						
	Cultural								
	uprooting/burning infested/ infected plants,								
	clean seed,								
	crop rotation,								
	intercropping,								
	early planting,								
	early harvesting								
	Botanicals/Bio-control (natural enemies)								
	Resistant varieties		1.						
			2.						
	Physical control (traps)								
	Mechanical control (hand picking)								
	None (Doesn't control)								
	Other (specify)								
	Other								

Crop	Control method	Used (Yes/No)	Product name (Read it from the pesticide container)	Active Ingredient (Read it from the pesticide container)	Target pest or disease	Application freq. per season (Min & Max times, give range)	Quantity (mls/kg per Knapsack)	Knap-sack size (L)	Area sprayed (s)
**Sweetpotato**	Chemical insecticides		1.						
			2.						
	Chemical fungicides		1.						
			2.						
	Chemical herbicides		1.						
			2.						
	Cultural (specify)								
	uprooting/burning infested/ infected plants,								
	clean vines,								
	crop rotation,								
	intercropping,								
	early planting,								
	early harvesting)								
	Botanicals/Bio-control (natural enemies)								
	Resistant varieties		1.						
			2.						
	Physical control (traps)								
	Mechanical control (hand picking)								
	None (Doesn't control)								
	Other (specify)								
	Other								
**Banana**	Chemical insecticides		1.						
			2.						
	Chemical fungicides		1.						
			2.						
	Chemical herbicides		1.						

Crop	Control method	Used (Yes/No)	Product name (Read it from the pesticide container)	Active Ingredient (Read it from the pesticide container)	Target pest or disease	Application freq. per season (Min & Max times, give range)	Quantity (mls/kg per Knapsack)	Knap-sack size (L)	Area sprayed (s)
			2.						
	Cultural								
	uprooting/burning infested/ infected plants,								
	clean suckers,								
	crop rotation,								
	intercropping,								
	early planting,								
	early harvesting								
	Botanicals/Bio-control (natural enemies)								
	Resistant varieties		1.						
			2.						
	Physical control (traps)								
	Mechanical control (hand picking)								
	None (Doesn't control)								
	Other (specify)								
	Other								
**Cassava**	Chemical insecticides		1.						
			2.						
	Chemical fungicides		1.						
			2.						
	Chemical herbicides		1.						
			2.						
	Cultural								
	uprooting/burning infested/ infected plants,								
	clean cuttings,								
	crop rotation,								

Crop	Control method	Used (Yes/No)	Product name (Read it from the pesticide container)	Active Ingredient (Read it from the pesticide container)	Target pest or disease	Application freq. per season (Min & Max times, give range)	Quantity (mls/kg per Knapsack)	Knap-sack size (L)	Area sprayed (s)
	intercropping,								
	delayed planting, early planting,								
	early harvesting)								
	Botanicals/Bio-control (natural enemies)								
	Resistant varieties	1.							
		2.							
	Physical control (traps)								
	Mechanical control (hand picking)								
	None (Doesn't control)								
	Other								

Do you know of any natural enemies (parasitoids or predators) of insect pests?........If yes, give name of enemy & pest ....

How do you dispose-off infested and/or infected tubers, roots, plants?…………………1.Leave them in the field 2.Burn/Burry/roadside/river 3. Feeds the animals 4……………………

Do you have pests problems in potato storage?...............If yes, what kind of pests and damage?..

What are the losses due to pest problems (give % damage)?..

Do you apply chemical pesticides in **potato** storage?.............If yes, name the chemical used..

Have you ever heard of integrated pest management (IPM)?......... If yes, what do you know about IPM?

……

……

Part G: Impact of pests and diseases on potato production, processing, marketing and consumption

Please state the crop losses in yield, quality, trade you made in the previous harvest (1st cropping season) due to pests and diseases

Crop	Size of field (ares)	Actual yield (bags or Kg)	Expected yield (bags or Kg)	Quantity lost (bags or Kg)	Monetary Loss (RWF/BIF)
**Potato**					
**Sweetpotato**					
**Banana**					
**Cassava**					

Part H: Impact of pest and diseases on household food security

Please answer the following questions if they applied to your household in the last 12 months

	**Statement**	**TRUE**	**FALSE**
1	Pest and disease damage deprived my family members of enough/required food to eat throughout the year		
2	Pest and disease damage caused food to be very expensive/unaffordable for my family members throughout the year		
3	Someone of my family members fell sick as a result of using pesticides		
4	I have always sold my **potatoes** at a reduced price (below market value) due to pest or disease damage		
5	I have always sold my **sweetpotatoes** at a reduced price (below market value) due to pest or disease damage		
6	I have always sold my **bananas** at a reduced price (below market value) due to pest or disease damage		
7	I have always sold my **cassava** at a reduced price (below market value) due to pest or disease damage		
8	I didn't dispose of any **potatoes** when they became rotten/stale/ unfit for human consumption		
9	I didn't dispose of any **sweetpotatoes** when they became rotten/stale/ unfit for human consumption		
10	I didn't dispose of any **bananas** when they became rotten/stale/ unfit for human consumption		
11	I didn't dispose of any **cassava** when they became rotten/stale/ unfit for human consumption		
12	Have you stopped growing any variety of **potato** because it is very susceptible to pests and/or diseases		
13	Have you stopped growing any variety of **sweetpotato** because it is very susceptible to pests and/or diseases		
14	Have you stopped growing any variety of **banana** because it is very susceptible to pests and/or diseases		
15	Have you stopped growing any variety of **cassava** because it is very susceptible to pests and/or diseases		
16	Has there been any new outbreak of a pest or disease of **potato** in the last 10 years?		
17	Has there been any new outbreak of a pest or disease of **sweetpotato** in the last 10 years?		
18	Has there been any new outbreak of a pest or disease of **banana** in the last 10 years?		
19	Has there been any new outbreak of a pest or disease of **cassava** in the last 10 years?		
20	I didn't plant at all any **potatoes** in the subsequent season following serious crop damage by pests and diseases		
21	I didn't plant at all any **sweetpotatoes** in the subsequent season following serious crop damage by pests and diseases		
22	I didn't plant at all any **bananas** in the subsequent season following serious crop damage by pests and diseases		
23	I didn't plant at all any **cassava** in the subsequent season following serious crop damage by pests and diseases		
24	I planted less quantity of **potato** because pest and disease damaged most of my **seed potato**		
25	I planted less quantity of **sweetpotato** because pest and disease damaged most of my **sweetpotato** vines		
26	I planted less quantity of **banana** because pest and disease damaged most of my bananas		
27	I planted less quantity of **cassava** because pest and disease damaged most of my **cassava**		
28	I grow **potatoes** primarily for home use (food)		
29	I grow **sweetpotatoes** primarily for home use (food)		
30	I grow **banana** primarily for home use (food)		
31	I grow **cassava** primarily for home use (food)		

Part I: Pesticide use and handling

How do you avoid contact with pesticides during application?

Protective gear	Yes/No	Precaution	Yes/No	Precaution	Yes/No
Wear gloves		Wear face and nose mask		Take a bath after spraying	
Wear gumboots		Wear long sleeved shirt		Observe wind direction while spraying	
Wear a hat		Wear waterproof jacket		Use a knapsack sprayer	
Wear goggles		Wear waterproof pants		Other (specify)	

If none, why don't you apply any preventive measure?...

Have you experienced any of the following after pesticide applications?

Effect	Yes/ No	Effect	Yes/ No	Effect	Yes/ No	Effect	Yes/ No	Effect	Yes /No
Skin itching		Coughing		Dizziness		Stomach ache		Tears	
Skin burning		Flu		Headache		Excess salivation		Burning eyes	
Heavy sweating		Breathing difficulties		Death of domestic animal		Nausea		Blurry vision	
				Human death/self-poisoning		Vomiting		Reddened eyes	

Can you tell how much money you have spent on treatment of pesticide poisoning related sickness in the last 12 months?..RWF/BIF

Where did you buy the chemical pesticides (insecticides+fungicides)? ....1. Agro vet shops 2. General merchandise shops 3.Weekly market 4.other…………

What is the pack size (Litres or Kg) of pesticides you bought in the last season (i) Smallest pack…………………….……… largest

pack/vol…………………………………….Kg or L

For how many years have you been applying pesticides in **potato** fields? ....Has the frequency of pesticide application increased in the last 10

years?................

For how many years have you been applying pesticides in **potato** stores? ....Has the frequency of pesticide application increased in the last 10

years?................

For how many years have you been applying pesticides in **sweetpotato** fields? ....Has the frequency of pesticide application increased in the last 10 years?...............

For how many years have you been applying pesticides in **banana** plantations? ....Has the frequency of pesticide application increased in the last 10 years?...............

For how many years have you been applying pesticides in **cassava** fields? ....Has the frequency of pesticide application increased in the last 10 years?...............

How do you know which pesticide to buy or apply?.............................(1.Seller 2.Another farmer 3. Own experience 4. Other...)

How do you know the doses of pesticides to use?.............................. (1.Seller 2.Another farmer 3. Can read 4. Other..)

Do you follow a fixed timetable to apply pesticides……….or do you only spray AFTER you have seen pest damage or disease signs?.......................................

Were pesticide sold in labelled containers?.........................If yes did you read and understand the pesticide label?..

Do you know how to tell the toxicity level of a pesticide from its label?............................If yes, let the farmer show you the sign on the container

Do you know any negative effect resulting from pesticide use on the environment?.............If yes, mention the effects …

………

....

Do you have knowledge about other methods of pest control?...

....

Part J: Social Capital and Networking

Have any member of your HH been involved in formal and/or farmer organizations in the last 3 years?...

Family code (See Part A)	Type of group: **Codes A**	Three most important group functions: **Codes B**		
		1st	2nd	3rd

Codes A	Codes A	Codes B	Codes B	Codes B
1. Farmers' cooperative/union<br>2. Farmers' village group<br>3. Faith based organization<br>4. NGO	5. Women's Association<br>6. Savings and Credit group<br>7. Other (Specify)	1. Produce marketing<br>2. Input supply<br>3. Savings & Credit	4. Credit provision<br>5.Labour sharing<br>6. Tree planting	7. Soil & water conservation<br>8. Other (Specify)

Part K: Crop production techniques and productivity

How much land do you own in ares?...

Total land cropped (ares)	Owned land (ares)	Owner (Family code)	Borrowed land (ares)	Owner (Family code)	Rented land (ares)	Owner (Family code)	.
1st Season (Mar-Jul)							
2nd Season (Aug-Dec)							

Which crops did you grow on your farm in the 1st and 2nd season? (Begin with crops planted on the largest area)

**Season 1**			**Season 2**		
**Crop**	Planted area (ares)	Crop owner (family code)	**Crop**	Planted area (ares)	Crop owner (family code)

Do you intercrop **potato**?.............................. If yes, with which crops?...

Do you intercrop **sweetpotato**?.............................. If yes, with which crops?...

Do you intercrop **banana**?.............................. If yes, with which crops?...

Do you intercrop **cassava**?.............................. If yes, with which crops?...

For how many years have you been growing **potato**? Man (Husband)..Woman (Wife)...

For how many years have you been growing **sweetpotato**? Man (Husband)..Woman (Wife)...

For how many years have you been growing **banana**? Man (Husband)..Woman (Wife)..

For how many years have you been growing **cassava**? Man (Husband)..Woman (Wife)...

How many varieties of **potato** did you plant in the 1st season…………………………..and 2nd season..

How many varieties of **sweetpotato** did you plant in the 1st season…………………………..and 2nd season...

How many varieties of **banana** are in the different plots? Plot 1…………………….Plot 2……………………………..plot 3………………………Plot 4...................................

How many varieties of **cassava** did you plant in the 1st season…………………………..and 2nd season..

For the last season, please state the following for **potato**

Name of potato variety	Planted area (ares)	Field owner (family code)	Source of seed (codes A) if more than one source, please specify percentage from each source	Quantity of seed (Kg or bags) if more than one source, please specify how much per source	Cost of 100kg seed (for each type of seed source)	Yield (Kg or bags)	Quantity sold (Kg or bags)	Price per bag (RWF/BIF)	Quantity used for food (Kg or bags)	Quantity kept for seed (Kg or bags)

Codes A<br>1.Govt Agric Extension	2.Own seed saved from previous season<br>3.NGO	4.Agric research org (NARO)<br>5.Another farmer		6. Agro-dealer<br>7. other...

For the last season, please state the following for **sweetpotato**

Name of sweetpotato variety	Planted area (in ares)	Field owner (family code	Source of vines (codes A) if more than one source, please specify percentage from each source	Quantity of vines (bags) if more than one source, please specify how much per source	Cost of 100kg bag (RWF/BIF)	Yield (Kg or bags)	Quantity sold (Kg or bags)	Price per bag (RWF/BIF)	Quantity used for food (Kg or bags)	Quantity kept for seed (Kg or bags)

Codes A<br>1.Govt agric Extension	2.Own seed saved from previous season<br>3.NGO	4.Agric research org (NARO)<br>5.Another farmer		6. Agro-dealer<br>7. other...

For the last season, please state the following for **banana**

Name of banana variety	Planted area (in ares)	Owner of the field (family code)	Source of suckers (codes A) if more than one source, please specify percentage from each source	No. of suckers (if more than one source, please specify how much per source)	Cost of 1 sucker (RWF/BIF)	Yield (Kg/ bunches)	Quantity sold (Kg or bunches)	Price per bunch/kg	Quantity used for food (Kg or bags)	Quantity kept for seed (Kg or bags)

Codes A<br>1.Govt Agric Extension	2.Own seed saved from previous season<br>3.NGO	4.Agric research org (NARO)<br>5.Another farmer		6. Agro-dealer<br>7. other……………………………………

For the last season, please state the following for **cassava**

Name of cassava variety	Planted area (in ares)	Owner of the field (family code)	Source of cuttings (codes A) if more than one source, please specify percentage from each source	Quantity of cuttings (bags) if more than one source, please specify how much per source	Cost of 100kg seed (RWF/BIF)	Yield (Kg or bags)	Quantity sold (Kg or bags)	Price per bag	Quantity used for food (Kg or bags)	Quantity kept for seed (Kg or bags)

Codes A<br>1.Govt Agric Extension	2.Own seed saved from previous season<br>3.NGO	4.Agric research org (NARO)<br>5.Another farmer		6. Agro-dealer<br>7. other……………………………………

Which of the following inputs do you use in **potato**? (DAP,NPK, UREA, Manure, Diathane, Ambush, Supergrow, Herbicides)

Field size (ares)	Fertilizer 1 …………………….		Fertilizer 2 …………		Organic manure …………		Insecticide 1 …………		Insecticide 2 …………		Fungicide 1 …………		Fungicide 1 …………		Others …………..
	Quantity (kg)	Cost (RWF/ BIF)	Qty (kg)	Cost (RWF/ BIF)	Qty (kg)	Cost (RWF/ BIF)	Vol. (L)	Cost (RWF/ BIF)	Vol. (L)	Cost (RWF/ BIF)	Qty (kg)	Cost (RWF/ BIF)	Qty (kg)	Cost (RWF/ BIF)	

Which of the following inputs do you use in **sweetpotato**? (DAP,NPK, UREA, Manure, Diathane, Ambush, Supergrow, Herbicides)

Field size (ares)	Fertilizer 1 ………………..		Fertilizer 2 …………		Organic manure …………		Insecticide 1 …………		Insecticide 2 …………		Fungicide 1 …………		Fungicide 1 …………	
	Quantity (kg)	Cost (RWF/ BIF)	Qty (kg)	Cost (RWF/BI F)	Qty (kg)	Cost (RWF/BI F)	Vol. (L)	Cost (RWF/BI F)	Vol. (L)	Cost (RWF/BI F)	Qty (kg)	Cost (RWF/BI F)	Qty (kg)	Cost (RWF/BI F)

Which of the following inputs do you use in **banana**? (DAP,NPK, UREA, Manure, Diathane, Ambush, Supergrow, Herbicides)

Field size (ares)	Fertilizer 1 ………………..		Fertilizer 2 …………		Organic manure …………		Insecticide 1 …………		Insecticide 2 …………		Fungicide 1 …………		Fungicide 1 …………	
	Quantity (kg)	Cost (RWF/ BIF)	Qty (kg)	Cost (RWF/BI F)	Qty (kg)	Cost (RWF/BI F)	Vol. (L)	Cost (RWF/BI F)	Vol. (L)	Cost (RWF/BI F)	Qty (kg)	Cost (RWF/BI F)	Qty (kg)	Cost (RWF/BI F)

Which of the following inputs do you use in **cassava**? (DAP,NPK, UREA, Manure, Diathane, Ambush, Supergrow, Herbicides)

Field size (ares)	Fertilizer 1 …………………		Fertilizer 2 ………..		Organic manure ………..		Insecticide 1 ………..		Insecticide 2 ………..		Fungicide 1 ………..		Fungicide 1 ………..	
	Quantity (kg)	Cost (RWF/ BIF)	Qty (kg)	Cost (RWF/BIF)	Qty (kg)	Cost (RWF/BIF)	Vol. (L)	Cost (RWF/BIF)	Vol. (L)	Cost (RWF/BIF)	Qty (kg)	Cost (RWF/BIF)	Qty (kg)	Cost (RWF/BIF)

During the last cropping season, how much money did you spend on paying for labor for the following tasks in **potato** production?

Field size (ares)	Land preparation	planting	weeding	chemical application	harvesting	Selling/ transport	Fertilizer application	Total labour cost (RWF/BIF)

During the last cropping season, how much money did you spend on paying for labor for the following tasks in **sweetpotato** production?

Field size (ares)	Land preparation	planting	weeding	chemical application	harvesting	Selling/ transport	Fertilizer application	Total labour cost (RWF/BIF)

During the last cropping season, how much money did you spend on paying for labor for the following tasks in **banana** production?

Field size (ares)	Land preparation	planting	weeding	chemical application	harvesting	Selling/ transport	Fertilizer application	Total labour cost (RWF/BIF)

During the last cropping season, how much money did you spend on paying for labor for the following tasks in **cassava** production?

Field size (ares)	Land preparation	planting	weeding	chemical application	harvesting	Selling/ transport	Fertilizer application	Total labour cost (RWF/BIF)

Part L: Market participation and access

Is there ready market for your **potatoes** in your village? 1. Seed potato ....2. Ware potato..

Is there ready market for your **sweetpotatoes** in your village? 1. Sweetpotato vines..................2. Sweetpotato roots..

Is there ready market for your **bananas** in your village? 1. Banana suckers/ macro-propagated plantlets.............................2. Banana bunches.........................

Is there ready market for your **cassava** in your village? 1. Cassava cuttings..2. Cassava roots..

From your house, what is the distance to (i) the nearest market/store……………………….km (ii) main road…………………………….…km

Regarding planting material, fill the table below

	Season 1								Season 2							
crop	Seed source	Sold planting material (Yes/No)	Qty sold	Unit price	Who sold	Who controlled cash	Who bought	Place of sale	Seed source	Sold planting material (Yes/No)	Qty sold	Unit price	Who sold	Who controlled cash	Who bought	Place of sale
**potato**																
**potato**																
**sweetpotato**																
**sweetpotato**																
**banana**																
**banana**																
**cassava**																
**cassava**																

Seed source: 1=own seed saved from previous harvest, 2=seed company 3=other farmer, 4=farmers' group, 5=Extension Agents, 6. Other (specify)…………………

Who in the family sold or controlled cash: 1. Husband 2.Wife

Who bought: 1.Farmers group, 2.Consumer 3.Broker 4.Seed company, 5.GOVT AGRIC EXTENSION)

Place of sale: (1.Onfarm, 2.Village market/store, 3.Main/town market, 4.Other(specify)……………………………………

Part M: Gender roles in crop production

Referring to the last cropping **YEAR**, who in the family decided or did the following activities regarding **potato**?

**Activity**	**Season 1**		**Season 2**	
	Who in the family decided (family code)	Who in the family worked or paid labor costs (family code)	Who in the family decided (family code)	Who in the family worked paid labor costs (family code)
Land preparation				
Bought inputs				
Selected the variety to plant				
Planted				
Weeded				
Applied Fungicides/insecticides				
Harvested				
Transported				
Processed				
Sold				

Referring to the last cropping **YEAR**, who in the family decided or did the following activities regarding **sweetpotato**?

**Activity**	**Season 1**		**Season 2**	
	Who in the family decided	Who in the family worked paid labor costs	Who in the family decided	Who in the family worked paid labor costs
Land preparation				
Bought inputs				
Selected the variety to plant				
Planted				
Weeded				
Applied Fungicides/insecticides				
Harvested				
Transported				
Processed				
Sold				

Referring to the last cropping **YEAR**, who in the family decided or did the following activities regarding **banana**?

**Activity**	**Season 1**		**Season 2**	
	Who in the family decided	Who in the family worked paid labor costs	Who in the family decided	Who in the family worked paid labor costs
Land preparation				
Bought inputs				
Selected the variety to plant				
Planted				
Weeded/mulched				
Applied Fungicides/insecticides /manure				
Harvested				
Transported				
Processed				
Sold				

Referring to the last cropping **YEAR**, who in the family decided or did the following activities regarding **cassava**?

**Activity**	**Season 1**		**Season 2**	
	Who in the family decided	Who in the family worked paid labor costs	Who in the family decided	Who in the family worked paid labor costs
Land preparation				
Bought inputs				
Selected the variety to plant				
Planted				
Weeded				
Applied Fungicides/insecticides				
Harvested				
Transported				
Processed				
Sold				

Part N: Access to extension services, credit and inputs

Has your family received any training in pest and disease management of **potato**?……… If yes, who in the family was trained ……………………..

Has your family received any training in pest and disease management of **sweetpotato**?……… If yes, who in the family was trained ………………..

Has your family received any training in pest and disease management of **banana**?……… If yes, who in the family was trained ……………………..

Has your family received any training in pest and disease management of **cassava**?……… If yes, who in the family was trained ……………………..

Did any agricultural extension officer get in contact with your family during the last 12 months?............. If yes, who in the family was contacted.............

How many times (days) was your family contacted in the last 12 months?...

What services did your family receive from the extension worker in the last cropping season?

……

……

……

What are your extension needs?

1………

2………

3………

In a year, how often do you receive diseases and pest control information/advice from the following sources? (see codes below)

**Information source**	**Time when advice is received**	**Number of times advice is received**	**Crop(s) for which advice is received**	**Was the advice effective (Yes/No)**
Neighbor/friend/relative				
Extension agent				
Agro-dealer				
NGO				
Radio/Tv				
Agricultural research organizations				
Middle men/produce buyers				
Agricultural shows/farmer field schools/field days				
Local farmers' group				
Farmer cooperative/ society				
Other (specify)_______________				

Codes: 1. Never, 2. Only when the disease or pest appears in my field, 3. Only when the disease or pest appears in my neighbor's field , 4. Every season, does not matter if the disease or pest is present , 5. Other (specify)……………………………….

Has any member of your household received any credit in the last 12 months?...................If yes, who in the family received credit.................................

If NOT, give reasons (1.No need 2.Risky 3.High interest rates 4. No banks nearby 5.No income to service the loan 6.Short repayment time 7.Other …………)

……

………I

f you received credit, how much did you receive and for what purpose?

**Amount of credit (RWF/BIF)**	**Credit source**	**Reason for getting credit**	**Who got the credit**

Credit sources: 1. Input dealer 2. Neighbors/friends/family 3.Private money lender 4. Commercial bank 5. Farmer's cooperative 6.Women's group 7. Other (specify)

Thank you for your time
